# Effects of naturally oxidized corn oil on inflammatory reaction and intestinal health of broilers

**DOI:** 10.1016/j.psj.2021.101541

**Published:** 2021-10-13

**Authors:** Yuqing Zhang, Tahir Mahmood, Zhenhai Tang, Yuqin Wu, Jianmin Yuan

**Affiliations:** ⁎ State Key Laboratory of Animal Nutrition, College of Animal Science and Technology, China Agricultural University, Beijing 100193, China; † Adisseo Animal Nutrition, Dubai 00000, United Arab Emirates

**Keywords:** oxidized oil, inflammation, intestinal barrier, intestinal microbiota, broiler chicken

## Abstract

This study was conducted to investigate the effects of naturally oxidized corn oil on the inflammatory reaction and intestinal health of broilers. Total 450, one-day-old Arbor Acres male broilers were randomly divided into 5 treatments with 6 replicate cages (15 birds in each replicate cage). The dietary treatment array consisted of the varying ratio of nonoxidized corn oil to naturally oxidized corn oil from 0:100, 25:75, 50:50, 75:25, and 100:0, respectively. The experimental period was 42 d. Serum, jejunum, and contents of cecum samples were taken at the age of 42 d of broilers. The results showed no significant difference in the body weight gain (**BWG**) with a different proportion of oxidized corn oil compared with the 0% oxidized oil group on d 42. The feed intake (**FI**), the concentration of immunoglobulin G (**IgG**), interferon-γ (**IFN-γ**), and interleukin-10 (**IL10**) in serum showed a significant quadratic response with the increase of oxidized oil concentration on d 42. The serum's concentration of IgG, IFN-γ, and IL-10 reached the highest value at 75% oxidized corn oil. In addition, the mRNA expression levels of interleukin-1β (**IL-1β**), IFN-γ, nuclear factor kappa B (**NF-κB**), tumor necrosis factor α (**TNF-α**), and myeloid differentiation factor-88 (**MyD88***)* in the jejunum were significantly affected by different proportions of oxidized corn oil, and the gene expression levels were highest at 75% oxidized corn oil on d 42. The mRNA expression of Bcl2-associated X (**Bax**) in the jejunum showed a significantly quadratic curve with the increase of oxidized oil concentration, and its gene expression was the highest after adding 50% oxidized corn oil according to the regression equation on d 42. The villus height/crypt depth and goblet cells of jejunum decreased linearly with the increasing proportion of oxidized corn oil and reached the lowest point after adding 100% oxidized corn oil on d 42. The β diversity showed the remarkable differentiation of microbial communities among 5 groups, and the microbial community of the 0% oxidized oil group was significantly separated from that of 75 and 100% oxidized oil groups in the cecum. Taken together, these results showed that a low dose of naturally oxidized corn oil is not harmful to the growth of broilers, while a high dose of oxidized corn oil will trigger the inflammatory response and adversely affect the gut health of broilers.

## INTRODUCTION

The gut is not only a major site for digestion and absorption of nutrients but also a selective barrier against toxins, pathogens, and antigens from the luminal environment (Ling et al., 2016). However, gut health is affected by numerous factors, including pathogens, environment, antinutritional factors, and peroxides (Mishra and Jha, 2019). Oil is often added to poultry diets to meet energy needs and for better growth performance. Due to high digestibility and efficient utilization, vegetable oil, for instance, corn oil, is routinely used in the animal feed industry. However, a higher proportion of unsaturated fatty acids is easily oxidized to produce a large number of free radicals and potentially cytotoxic fatty acid degradation products (Kanner, 2007).

Moreover, most hydroperoxides break down into hydroxides, epoxides, and aldehydes in the stomach. Aldehydes can enter the small intestine and be partially absorbed into the body, which can cause oxidative stress (Suomela et al., 2005). Oxidative stress can inhibit the signal transduction of associated intestinal lymphocytes and promote endotoxin and inflammatory response (Cui et al., 2009). Research has shown that oxidized diets enhance plasma inflammatory markers and activate nuclear factor kappa B (**NF-κB**) in the small intestine, and consumption of oxidized n-3 PUFA results in 4-HHE accumulation in blood after its intestinal absorption and triggers oxidative stress and inflammation in the upper intestine (Awada et al., 2012). A recent study showed that oxidized fish oil could cause intestinal dysbiosis, barrier dysfunction, and hepatic inflammation mediated by gut-derived endotoxin (Feng et al., 2020). Importantly, induction of oxidative stress can be associated with intestinal inflammation and changes in host microbiota. It has been found that oxidized fish oil improved the microbial ability to synthesize lipopolysaccharide, which was the main component of the cell wall of gram-negative bacteria and a highly proinflammatory toxin (Maldonado et al., 2016).

Previous research was mostly furnished by high-temperature oxidized oil. However, the mechanism of high-temperature oxidized oil is the same as that of naturally oxidized oil at room temperature, which belongs to free radical reaction, and the products after oxidation are different (Kaleem et al., 2015). After natural oxidation, oil mainly produces hydroperoxide, and hydroperoxides are unstable and quickly produce small molecules such as aldehydes, ketones, and acids (Zhang et al., 2012). However, it is still unclear whether naturally oxidized corn oil has adverse effects on broilers and whether it can be used to reduce costs in poultry production. Therefore, we conducted this study to explore the effects of naturally oxidized corn oil on the inflammatory response and intestinal health of broilers to provide insight on the potential usage of oxidized oil in poultry production.

## MATERIALS AND METHODS

### Ethics Statement

All animal procedures were performed in accordance with the Guidelines for Care and Use of Laboratory Animals of China Agricultural University and approved by the Animal Ethics Committee of China Agricultural University, and all experiments followed institutional guidelines (approval number: AW04129102-1-1).

### Preparation of Oxidized Oil

Fresh corn oil was purchased from the crude oil factory. Part of corn oil was put in an open container and was prepared under the condition of 30 to 40°C, with outdoor sunlight (average light intensity:1,000 Lux) and oxygen for 60 d as naturally oxidized oil, and the other stored in a barn under 20 to 30°C without light and oxygen as nonoxidized oil. The peroxide value of oxidized oil and nonoxidized oil was 68.4 meq/kg and 9.8 meq/kg, respectively.

### Birds, Diets, and Experimental Design

A total of 450, one-day-old male Arbor Acres broilers were randomly assigned to 5 dietary treatments (each involving 6 replicate cages with 15 birds each). The treatment groups were 0, 25, 50, 75, and 100% naturally oxidized oil in place of nonoxidized oil, respectively.

The basal diet was formulated to meet or exceed the nutrient requirement for broilers recommended by NRC (NRC 1994). The experimental period was 42 d. The ingredient and nutrient compositions of the basal diets for the starter (0–21 D) and finisher (22–42 D) phases are shown in Table 1. The starter diet was pelleted and crumbled. While the finisher diet was fed in pelleted form. Water was provided ad libitum using a nipple-type drinker. The room temperature was maintained at 33 to 35°C during the first 3 D, followed by a reduction to 28°C to 30°C during the next 2 wk and 22 to 25°C for the remainder of the trial. A standard lighting regime was followed: 23 h of light and 1 h of darkness for the first 8 D, followed by 20 h of light and 4 h of darkness from d 9 until the end of the trial.

**Table 1:** Ingredients and composition of the basal experimental diets.

Ingredients, %	Starter diet	Finisher diet
Corn	51.50	56.60
Soybean meal	30.58	26.10
Corn DDGS	5.00	5.00
Corn gluten meal	4.00	4.00
Corn oil^1^	2.70	4.70
Wheat flour	2.00	0.00
Dicalcium phosphate	1.64	1.20
Limestone	1.27	1.20
Salt	0.35	0.35
Trace mineral premix^2^	0.20	0.2
Vitamin premix^3^	0.03	0.02
Choline chloride (50%)	0.20	0.20
DL-Methionine	0.24	0.20
L-Lysine sulfate	0.26	0.20
Antioxidant	0.02	0.02
Phytase	0.01	0.01
Nutrient composition, %^4^		
ME (kcal/kg)	2950	3146
CP, %	22.50	20.50
Lysine, %	1.30	1.12
Methionine, %	0.59	0.52
Methionine+Cysteine, %	0.94	0.85
Calcium, %	1.00	0.80
NPP, %	0.45	0.32

1 The same percentage of corn oil was used in different treatment groups. The treatment groups were as follows: the ratio of nonoxidized corn oil to naturally oxidized oil was 0:100, 25:75, 50:50, 75:25, 100:0, respectively.

2 The trace mineral premix provided the following per kg of diets: Cu,16 mg (as CuSO_4_•5H_2_O); Zn, 110 mg (as ZnSO_4_); Fe, 80 mg (as FeSO_4_•H_2_O); Mn, 120 mg (as MnO); Se, 0.3 mg (as Na_2_SeO_3_); I, 1.5 mg (as KI); Co, 0.5 mg.

3 The vitamin premix provided the following per kg of diets: vitamin A, 10,000 IU; vitamin D_3_, 2,400 IU; vitamin E, 20 mg; vitamin K_3_, 2 mg; vitamin B_1_, 2 mg; vitamin B_2_, 6.4 mg; VB_6_, 3 mg;VB_12_, 0.02 mg; biotin, 0.1 mg; folic acid, 1 mg; pantothenic acid,10 mg; nicotinamide, 30 mg.

4 All the nutrient levels are calculated values.

### Performance Parameters

Chickens and feed were weighed by cage (replicate) on the day of hatch and on d 42. Body weight gain (**BWG**), Feed intake (**FI**), and feed conversion ratio (**FCR**) were calculated for the whole experiment. BWG = (final chicken weight (kg) / final chicken number) - (initial chicken weight (kg) / initial chicken number); FI = (total feed intake (kg) - dead chicken feed intake (kg)) / number of live chickens; FCR = (initial feed weight (kg) − residual feed weight (kg)) / (dead chicken weight (kg) + live chicken weight (kg)); Feed intake of dead chicken = FCR at death × dead chicken weight (kg).

### Sample Collection

On d 42, 6 birds (1 bird per cage) of each treatment were randomly selected. After collecting 4 mL blood sample from the wing vein, chickens were killed by intracardial administration of sodium pentobarbital (30 mg/kg of body weight). Serum was harvested after centrifuging the blood at 4°C at 3,000 *g* for 15 min and stored at −80°C until further analysis. Samples of the jejunum (1 cm) were excised for morphological analysis. In addition, three pieces of jejunum (0.5 cm) samples were collected and cleaned with sterile PBS, snap frozen in liquid nitrogen, and then transferred to −80°C for cryopreservation to extract RNA. The contents of the cecum were collected aseptically, snap-frozen, and stored at −80°C for 16S rRNA sequencing analysis.

### Serum Immunoglobulin Levels

The serum immunoglobulin A (**IgA**) and immunoglobulin G (**IgG**) levels were determined using a commercial ELISA kit (IgA, cat# ml002792-2; IgG, cat# ml042771-2) according to the manufacturer's recommended protocol (Shanghai enzyme union biology Co., Ltd, Shanghai, China).

### Serum Inflammatory Factor Concentration

The serum interferon-γ (**IFN-γ**), tumor necrosis factor α (**TNF-α**), interleukin-1β (**IL-1β**), interleukin-6 (**IL-6**), interleukin-8 **(IL-8**), and interleukin-10 (**IL-10**) concentration were determined using a commercial ELISA kit (IFN-γ, cat# ml042758-2; TNF-α, cat# ml002790-2; IL-1β, cat# ml059835-2; IL-6, cat# ml042757-2; IL-8, cat# ml059840-2; IL-10, cat# ml059830-2) according to the manufacturer's recommended protocol (Shanghai enzyme union biology Co., Ltd).

### RNA Isolation and Quantitative Reverse Transcription Polymerase Chain Reaction

The RNA was extracted from the jejunum using the Eastep Super Total RNA Extraction Kit (Promaga Co., Shanghai, China). RNA quantity was measured using the Nanodrop (Thermo Fisher, Waltham, MA). Then, total RNA was reversed transcribed to cDNA using the PrimeScrip RT reagent Kit with gDNA Eraser Perfect Real-Time (Takara Biomedical Technology, Beijing, China), and the gene expression levels were determined using the SYBR Premix Ex Taq Tli RNaseH Plus (Takara Biomedical Technology) according to the product protocols. Primer sequences of the β-actin, IL-1β, IFN-γ, IL-8, TNF-α, toll-like receptor-4 (**TLR-4**), nuclear factor kappa B (**NF-κB**), myeloid differentiation factor88 (**MyD88**); B-cell lymphoma-2 (**Bcl-2**), cysteinyl aspartate specific proteinase-3 (**Caspase-3**), Bcl2-associated X (**Bax**), occludin, claudin-1, zonula occluden-1 (**ZO-1**), mucin-2 in the jejunum are listed in Table 2. All the gene sequences were quoted from NCBI. All the measurements were carried out in triplicate (N = 6, the cage was used as experimental unit), and the average values were calculated. Relative expression levels of different genes were normalized to the expression of β-actin using the 2 ^−∆∆CT^ method.

**Table 2:** Primers used in real-time quantitative PCR.

Gene	Gene bank ID	Primer sequence (5’-3′)
β-actin	NM_205518.1	F:CCACCGCAAATGCTTCTAAAC R:AAGACTGCTGCTGACACCTTC
IL-1β	XM_015297469.1	F:ACTGGGCATCAAGGGCTA R:GGTAGAAGATGAAGCGGGTC
IFN-γ	NM_205149.1	F: CTTCCTGATGGCGTGAAGA R: GAGGATCCACCAGCTTCTGT
IL8	NM_205018.1	F:ATGAACGGCAAGCTTGGAGCTG R:TCCAAGCACACCTCTCTTCCATCC
TNF-α	NM_204267.1	F: CCCCTACCCTGTCCCACAA R: TGAGTACTGCGGAGGGTTCAT
TLR-4	NM_001030693.1	F:GGATCTTTCAAGGTGCCACA R: CAAGTGTCCGATGGGTAGGT
NF-κB	NM_205129.1	F:ACCCCTTCAATGTGCCAATG R:TCAGCCCAGAAACGAACCTC
MyD88	NM_001030962.3	F: TGCAAGACCATGAAGAACGA R: TCACGGCAGCAAGAGAGATT
Bcl-2	NM_205339.2	F:GAGTTCGGCGGCGTGATGTG R:CTCGGTCATCCAGGTGGCAATG
Caspase-3	NM_204725.1	F:TACCGGACTGTCATCTCGTTCAGG R:ACTGCTTCGCTTGCTGTGATCTTC
Bax	XM_204725	F:TCCTCATCGCCATGCTCAT R:CCTTGGTCTGGAAGCAGAAGA
occludin	NM_205128.1	F: ACGGCAGCACCTACCTCAA R: GGGCGAAGAAGCAGATGAG
claudin-1	NM_001013611.2	F: CATACTCCTGGGTCTGGTTGGT R: GACAGCCATCCGCATCTTCT
ZO-1	NM_001301025.3	F: CTTCAGGTGTTTCTCTTCCTCCTC R: CTGTGGTTTCATGGCTGGATC
mucin-2	NM_001318434.1	F: CCCTCACCCAGCCCGACTTC R: GCCGTTGGTGGAGGTGTTACAG

Abbreviations: F, forward; R, reverse.

### Intestinal Histomorphology

The jejunum was fixed in 4% paraformaldehyde solution for more than 24 h. After dehydration and infiltration with solidified paraffin wax, the samples were embedded and cut at 5 μm with a sledge microtome. Hematoxylin-eosin and periodic acid Schiff stain were used to measure intestinal villi morphology and the number of goblet cells, respectively. The villus height (from the tip of the villus to the crypt opening) and the crypt depth (from the base of the crypt to the crypt opening) were measured from 10 randomly selected villi and the associated crypt, with one section per chicken at 40 × magnification. The number of goblet cells was measured in the same 10 randomly selected villi according to the method (Röhe et al., 2020).

### Cecal Microbiota Populations

The cecal contents of broilers were isolated to extract the total microbial community DNA using DNeasy PowerSoil Kit (QIAGEN, Inc., Netherlands). The quantity and quality of extracted DNAs were measured using a NanoDrop ND-1000 spectrophotometer (Thermo Fisher Scientific) and agarose gel electrophoresis, respectively. PCR amplification of the bacterial 16S rRNA genes V3–V4 region was performed using the forward primer 338F (5’-ACTCCTACGGGAGGCAGCA-3′) and the reverse primer 806R (5’-GGACTACHVGGGTWTCTAAT-3′) (Chang et al., 2018). The PCR reaction system carried out the following procedure: initial denaturation at 98°C for 2 min, denaturation at 98°C for 15 s, annealing at 55°C for 30 s, extension at 72°C for 30 s, repeated for 25 cycles, final extension at 72°C for 5 min, and hold at 10°C. PCR amplicons were purified with Vazyme VAHTSTM DNA Clean Beads (Vazyme, Nanjing, China) and quantified using the Quant-iT PicoGreen dsDNA Assay Kit (Invitrogen, Carlsbad, CA). After the individual quantification step, amplicons were pooled in equal amounts, and paired-end 2 × 300 bp sequencing was performed using the Illumina MiSeq platform with MiSeq Reagent Kit v3 at Shanghai Personal Biotechnology Co., Ltd (Shanghai, China). Microbiome bioinformatics was performed with QIIME2 with slight modification according to the official tutorials (https://docs.qiime2.org/2019.4/tutorials/). Sequences were then quality filtered, denoised, merged, and chimera removed using the DADA2 plugin. Sequences after processed were clustered at 100% sequence identity to generate amplicon sequence variants (**ASVs**), and each amplicon sequence variant (**ASV**) was annotated using the Greengenes 16S rRNA database (http://greengenes.secondgenome.com/). Alpha diversity indices, such as Chao1 index, Shannon index, Simpson index, were calculated using the ASV table in QIIME2. Beta diversity analysis was performed to investigate the structural variation of microbial communities across samples and visualized via principal coordinate analysis (**PCoA**). The significance of differentiation of microbiota structure among groups was assessed by Permutational multivariate analysis of variance (**PERMANOVA**). Linear discriminant analysis effect size (**LEfSe**) was performed to detect differentially abundant taxa across groups using the default parameters. All figures were drawn in R language. The microbiota reads were submitted to the NCBI-SRA database under accession number PRJNA745584.

### Statistical Analysis

Data were statistically analyzed by one-way ANOVA variance analysis using SPSS 20.0 for Windows (SPSS Inc. Chicago, IL). All data were tested for homogeneity of variances using Levene's test. We analyzed different proportions of naturally oxidized corn oil levels in the basal diet to get linear or quadratic responses. The significance among the groups was identified using the Duncan test for multiple comparisons. Results were presented as mean values with pooled standard errors. A *P* value of <0.05 was considered to be statistically significant, and *P* values between 0.05 and 0.10 were classified as trends. The regression equation is calculated by Microsoft Excel (2016).

## RESULTS

### Effects of Different Proportions of Naturally Oxidized Corn Oil on Growth Performance of Broilers

There was no significant difference in BWG with a different proportion of oxidized corn oil compared with OXA. The BWG of the 50% oxidized corn oil group was significantly higher than that of the 25% and 100% oxidized corn oil group (*P* < 0.05). FI (y = -0.93x^2^ + 1.05x + 3.82, *R*^2^ = 0.644, *P* < 0.05) had a significant quadratic relationship with the increase of the proportion of oxidized corn oil, and FI reached the maximum when 50% oxidized oil replaced the oil according to the regression equation (Table 3).

**Table 3:** Effects of different proportions of naturally oxidized corn oil on growth performance of broilers.

Parameter	OXA	OXB	OXC	OXD	OXE	SEM	*P*-value	*P*-linear	*P*-quadratic
BWG^1^/kg	2.31^ab^	2.19^b^	2.43^a^	2.32^ab^	2.24^b^	0.02	0.034	0.962	0.155
FI^2^/kg	3.88^b^	3.90^b^	4.14^a^	4.18^a^	3.89^b^	0.03	0.006	0.187	0.004
FCR^3^	1.71	1.78	1.72	1.79	1.73	0.01	0.291	0.536	0.243

Abbreviations: OXA, basal diet with 0% naturally oxidized oil; OXB, basal diet with 25% naturally oxidized oil; OXC, basal diet with 50% naturally oxidized oil; OXD, basal diet with 75% naturally oxidized oil; OXE, basal diet with 100% naturally oxidized oil.

ab Different letters in the same row indicate significant differences (*P* < 0.05), and the same letter means no significant difference (*P* > 0.05).

1 BWG, body weight gain, kg/bird.

2 FI, feed intake, kg/bird.

3 FCR, feed conversion ratio.

### Effects of Different Proportions of Naturally Oxidized Corn Oil on Serum Immune-Related Molecules of Broilers

Serum IgG (y = −265.90x^2^ + 247.27x + 329.34, *R*^2^ = 0.651, *P* < 0.05), IFN-γ (y = −10.93x^2^ + 10.36x + 6.83, *R*^2^ = 0.758, *P* < 0.05), and IL-10 (y = −11.28x^2^ + 10.59x + 10.07, *R*^2^ = 0.825, *P* < 0.05) showed a significantly quadratic curve with the increase of oxidized oil concentration (*P* < 0.05). The concentration of IgG, IFN-γ, and IL-10 in the serum reached the highest value when 75% oxidized oil added according to the regression equation. The serum IgA concentration of 100% oxidized corn oil group was significantly lower than that of other groups (*P* < 0.05; Table 4).

**Table 4:** Effects of different proportions of naturally oxidized oil on serum immune-related molecules of broilers.

Parameter	OXA	OXB	OXC	OXD	OXE	SEM	*P*-value	*P*-linear	*P*-quadratic
IgA (μg/mL)	43.97^a^	38.22^ab^	44.62^a^	44.72^a^	33.43^b^	1.33	0.012	0.077	0.074
IgG (μg/mL)	337.99^bc^	363.82^ab^	366.78^ab^	402.24^a^	295.49^c^	9.52	0.002	0.383	0.001
IFN-γ (pg/mL)	7.33^cd^	7.60^bc^	9.76^a^	8.97^ab^	5.93^d^	0.32	<0.001	0.393	<0.001
TNF-α (pg/mL)	10.98	11.27	10.47	9.99	10.86	0.26	0.624	0.438	0.522
IL-1β (pg/mL)	89.11	92.87	92.44	78.71	81.55	2.74	0.368	0.141	0.513
IL-6 (pg/mL)	4.81	3.96	4.36	4.11	3.76	0.12	0.055	0.020	0.711
IL-8 (pg/mL)	16.86	19.10	17.18	17.20	15.60	0.43	0.153	0.144	0.110
IL-10 (pg/mL)	10.43^bc^	11.38^ab^	12.37^a^	12.57^a^	8.98^c^	0.33	0.001	0.337	<0.001

Abbreviations: OXA, basal diet with 0% naturally oxidized oil; OXB, basal diet with 25% naturally oxidized oil; OXC, basal diet with 50% naturally oxidized oil; OXD, basal diet with 75% naturally oxidized oil; OXE, basal diet with 100% naturally oxidized oil.

abcd Different letters in the same row indicate significant differences (*P* < 0.05), and the same letter means no significant difference (*P* > 0.05).

### Effects of Different Proportions of Naturally Oxidized Oil on Jejunal Morphology

The VH (y = −1484.80x^2^ + 1278.00x + 879.43, *R*^2^ = 0.661, *P* < 0.05) of jejunum showed a significantly quadratic curve with increased oxidized oil concentration. The VH of jejunum reached the highest value after adding 50% oxidized oil and reached the lowest value after adding 100% oxidized oil. However, the CD of jejunum increased linearly, and the VH/CD and goblet cells of jejunum decreased significantly linearly with the increasing proportion of oxidized oil and reached the lowest point after adding 100% oxidized oil (*P* < 0.05; Table 5).

**Table 5:** Effects of different proportions of naturally oxidized oil on jejunal morphology of broilers.

Parameter	OXA	OXB	OXC	OXD	OXE	SEM	*P*-value	*P*-linear	*P*-quadratic
VH (μm)	942.38^b^	919.34^b^	1329.87^a^	945.94^b^	670.53^c^	50.76	<0.001	0.045	<0.001
CD (μm)	122.19^b^	188.70^ab^	214.44^a^	214.02^a^	210.12^a^	9.67	0.003	0.001	0.116
VH/CD	8.12^a^	4.94^bc^	6.19^b^	4.68^bc^	3.28^c^	0.38	<0.001	<0.001	0.698
Number of goblet cells (cells/ 100 μm)	11.70^a^	10.66^b^	9.33^c^	8.13^d^	8.43^d^	0.28	<0.001	<0.001	0.124

Abbreviations: OXA, basal diet with 0% naturally oxidized oil; OXB, basal diet with 25% naturally oxidized oil; OXC, basal diet with 50% naturally oxidized oil; OXD, basal diet with 75% naturally oxidized oil; OXE, basal diet with 100% naturally oxidized oil.

abcd Different letters in the same row indicate significant differences (*P* < 0.05), and the same letter means no significant difference (*P* > 0.05).

### Effects of Different Proportions of Oxidized Oil on mRNA Expression of Jejunum Inflammatory Cytokines and Apoptosis

Regression analysis showed that the mRNA expression levels of IL-1β, IFN-γ, NF-κB, TNF-α, and MyD88 in the jejunum were significantly affected by different proportions of oxidized oil, and the gene expression levels were the highest after adding 75% oxidized oil (*P* < 0.05). The mRNA expression of Bax in the jejunum showed a significantly quadratic curve with the increase of oxidized oil concentration (y = −0.82x^2^ + 0.67x + 1.03, *R*^2^ = 0.556, *P* < 0.05), and the gene expression was the highest after adding 50% oxidized oil instead of nonoxidized oil (Table 6).

**Table 6:** Effects of different proportions of naturally oxidized oil on relative mRNA expression of jejunum inflammation and apoptosis cytokines genes of broilers.

Parameter^1^	OXA	OXB	OXC	OXD	OXE	SEM	*P*-value	*P*-linear	*P*-quadratic
IL-1β	1.00^b^	1.45^ab^	1.84^ab^	2.22^a^	2.17^a^	0.09	0.047	0.568	0.109
IFN-γ	1.00^b^	0.99^b^	1.14 b	1.89^a^	1.12^b^	0.11	0.043	0.057	0.499
IL8	1.00	1.05	0.90	1.03	0.59	0.04	0.267	0.114	0.262
TNF-α	1.00^b^	1.23^ab^	1.21 ab	1.80^a^	1.30^ab^	0.04	0.045	0.39	0.073
TLR4	1.00	0.54	0.64	0.55	0.78	0.02	0.166	0.35	0.038
NF-κB	1.00^b^	1.28^ab^	1.03^b^	1.74^a^	0.97^b^	0.10	0.042	0.064	0.552
MyD88	1.00^b^	1.22^b^	1.45^ab^	1.94^a^	1.47^ab^	0.11	0.041	0.271	0.386
Bcl-2	1.00	1.11	0.66	0.73	0.74	0.13	0.397	0.15	0.637
Caspase-3	1.00	0.95	0.77	0.98	0.93	0.08	0.507	0.751	0.289
Bax	1.00^b^	0.91^b^	1.26^a^	1.17^ab^	0.94^b^	0.04	0.030	0.189	0.031

Abbreviations: Bax, Bcl2-associated X; Bcl-2, B-cell lymphoma-2; Caspase-3, cysteinyl aspartate specific proteinase-3; IL-1β, interleukin-1β; IFN-γ, interferon-γ; IL-8, interleukin-8; MyD88, myeloid differentiation factor88; NF-κB, nuclear factor kappa B; OXA, basal diet with 0% naturally oxidized oil; OXB, basal diet with 25% naturally oxidized oil; OXC, basal diet with 50% naturally oxidized oil; OXD, basal diet with 75% naturally oxidized oil; OXE, basal diet with 100% naturally oxidized oil; TNF-α, tumor necrosis factor α; TLR-4, toll-like receptor-4.

ab Different letters in the same row indicate significant differences (*P* < 0.05), and the same letter means no significant difference (*P* > 0.05).

1 Gene relative expression in different treatments of broilers (N = 6).

### Effects of Different Proportions of Oxidized Oil on mRNA Expression of Jejunal Barrier Protein

The mRNA expressions of occludin and mucin-2 in the jejunum were significantly affected by different proportions of oxidized oil (*P* < 0.05). In the 75% oxidized oil group, the mRNA expression levels of occludin and mucin-2 in jejunum were significantly lower than nonoxidized oil group (Table 7).

**Table 7:** Effects of different proportions of oxidized oil on mRNA expression of jejunal barrier protein-related genes of broilers.

Parameter^1^	OXA	OXB	OXC	OXD	OXE	SEM	*P*-value	*P*-linear	*P*-quadratic
occludin	1.00^a^	0.99^a^	0.75^b^	0.81^b^	0.89^ab^	0.02	0.013	0.360	0.277
claudin-1	1.00	1.60	0.85	0.72	0.64	0.12	0.577	0.987	0.857
ZO-1	1.00	0.69	0.66	0.63	0.71	0.07	0.124	0.07	0.056
mucin-2	1.00^ab^	0.81^b^^c^	1.17^a^	0.67^c^	0.82^b^^c^	0.04	0.002	0.066	0.541

Abbreviations: OXA, basal diet with 0% naturally oxidized oil; OXB, basal diet with 25% naturally oxidized oil; OXC, basal diet with 50% naturally oxidized oil; OXD, basal diet with 75% naturally oxidized oil; OXE, basal diet with 100% naturally oxidized oil; ZO-1, zonula occluden-1.

abc Different letters in the same row indicate significant differences (*P* < 0.05), and the same letter means no significant difference (*P* > 0.05).

1 Gene relative expression in different treatments of broilers (N = 6).

### Cecal Microbiota

**Figure 1:**
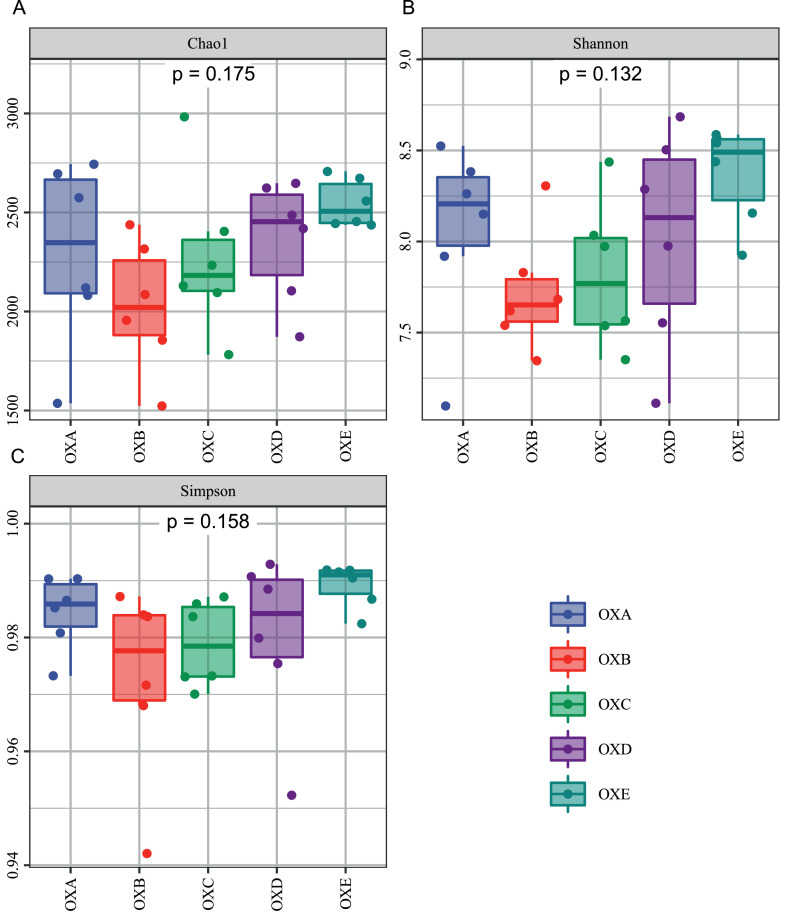
Effects of different proportions of naturally oxidized oil on the α-diversity of cecal microbiota. The α-diversity was evaluated by (A) Chao1 index, (B) Shannon index, and (C) Simpson index. Abbreviations: OXA, basal diet with 0% naturally oxidized oil; OXB, basal diet with 25% naturally oxidized oil; OXC, basal diet with 50% naturally oxidized oil; OXD, basal diet with 75% naturally oxidized oil; OXE, basal diet with 100% naturally oxidized oil.

**Figure 2:**
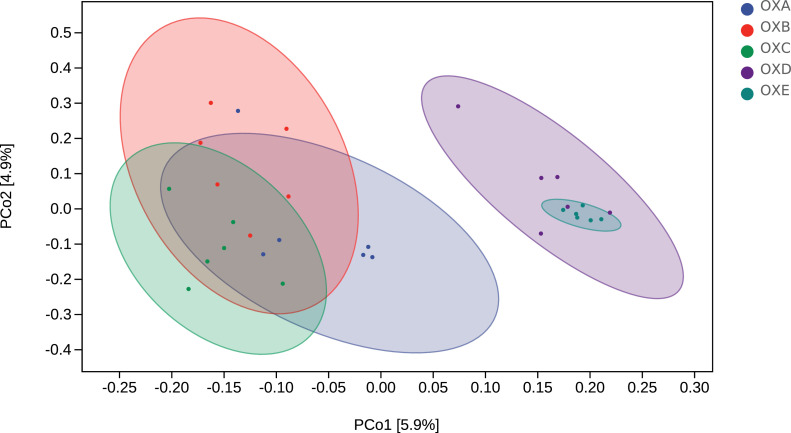
Effects of different proportions of naturally oxidized oil on the β-diversity of cecal microbiota. The β-diversity was evaluated by partial least squares discriminant analysis (PLS-DA). Abbreviations: OXA, basal diet with 0% naturally oxidized oil; OXB, basal diet with 25% naturally oxidized oil; OXC, basal diet with 50% naturally oxidized oil; OXD, basal diet with 75% naturally oxidized oil; OXE, basal diet with 100% naturally oxidized oil.

The average number of sequences in each treatment group was 122,085 for the 16S rRNA genes. The range of the rRNA sequence was 97,047 to 145,207. After screening these gene sequences, 59,731 valid sequences were obtained on average, accounting for 48.9% of the raw sequences. The range of valid rRNA sequence was 47,380 to 73,052. Alpha diversity indices: Chao1 index are suggesting the abundance of the community. Shannon and Simpson indexes are commonly used to measure the diversity of the community. With the addition of oxidized oil, the alpha diversity of the cecal microbiota was not significantly different between treatments (Figure 1, Table 8). The β diversity was analyzed by PERMANOVA, which showed the remarkable differentiation of microbial communities among the 5 groups (*P* < 0.05). The result was depicted via PCoA (Figure 2).

**Table 8:** Effects of different proportions of naturally oxidized oil on the α-diversity of cecal microbiota.

Parameter	OXA	OXB	OXC	OXD	OXE	SEM	*P*-value	*P*-linear	*P*-quadratic
Chao1	2,292.45	2,029.02	2,271.75	2,359.35	2,545.66	66.33	0.175	0.073	0.172
Shannon	8.05	7.72	7.81	8.01	8.36	0.08	0.132	0.115	0.037
Simpson	0.98	0.97	0.97	0.98	0.98	0.01	0.158	0.26	0.042

Abbreviations: OXA, basal diet with 0% naturally oxidized oil; OXB, basal diet with 25% naturally oxidized oil; OXC, basal diet with 50% naturally oxidized oil; OXD, basal diet with 75% naturally oxidized oil; OXE, basal diet with 100% naturally oxidized oil.

**Figure 3:**
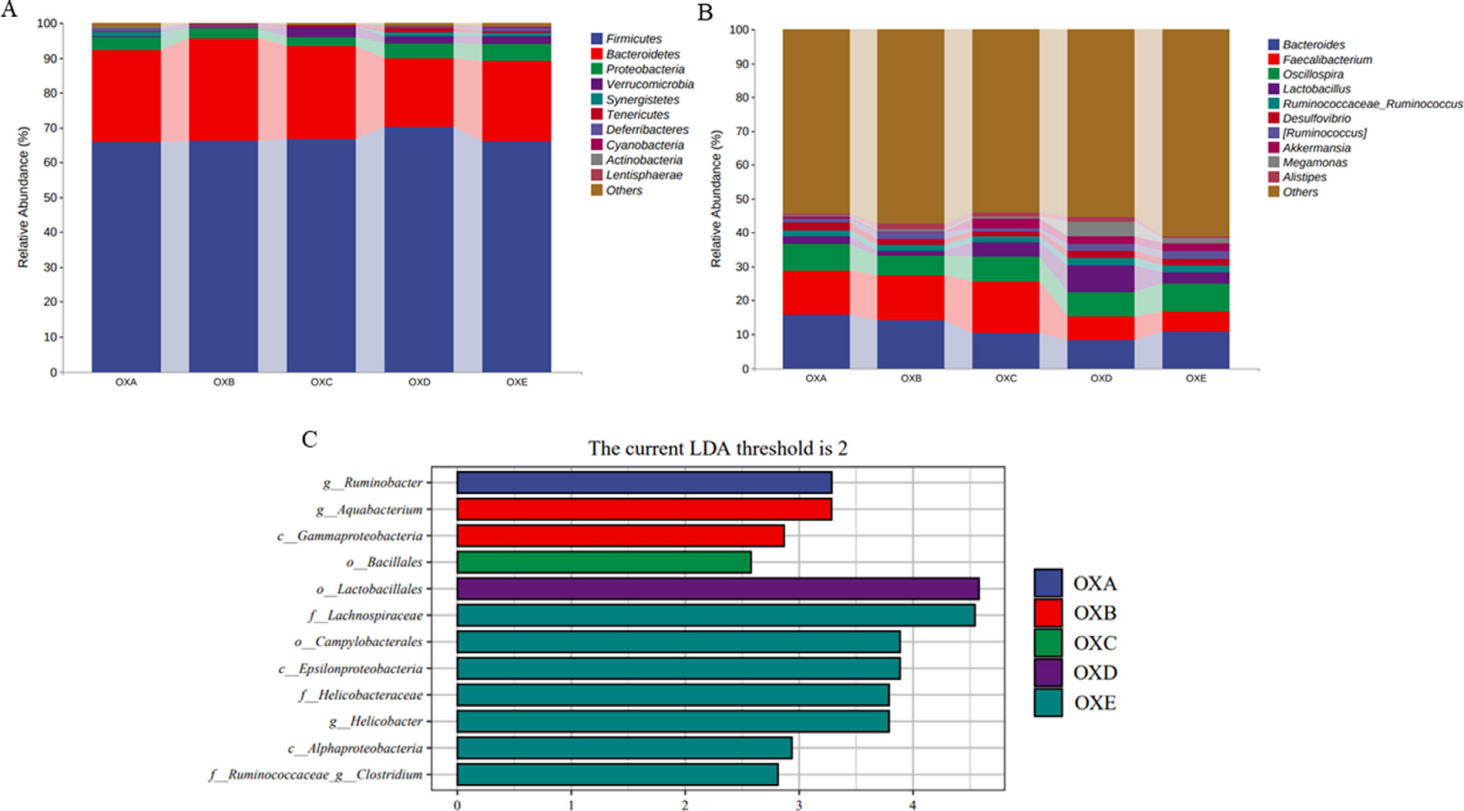
Effects of different proportions of naturally oxidized oil on cecal microbiota composition. Microbial community bar plot at the (A) phylum and (B) genus level. Lefse (LDA effect size) analysis (C) which the LDA scores ≥2. N = 6. Abbreviations: OXA, basal diet with 0% naturally oxidized oil; OXB, basal diet with 25% naturally oxidized oil; OXC, basal diet with 50% naturally oxidized oil; OXD, basal diet with 75% naturally oxidized oil; OXE, basal diet with 100% naturally oxidized oil.

At the phylum level, *Firmicutes, Bacteroidetes, Proteobacteria*, and *Verrucomicrobia* accounted for more than 90% of the total cecal microbiota. *Proteobacteria* had a significant quadratic curve relationship that first decreased and then increased with the increase of naturally oxidized oil (*P* < 0.05), and the relative abundance of *Proteobacteria* was the lowest when 50% oxidized oil was added (Figure 3A, Table 9). Among the top 10 genera, the relative abundance of *Lactobacillus* in the cecum was significantly affected by different proportions of oxidized oil (*P* < 0.05). The highest relative abundance of *Lactobacillus* was obtained when 75% oxidized oil was added (Figure 3B, Table 10).

**Table 9:** Effects of different proportions of naturally oxidized oil on cecal microbiota composition at the phylum level of broilers.

Parameter(%)	OXA	OXB	OXC	OXD	OXE	SEM	*P*-value	*P*-linear	*P*-quadratic
*Firmicutes*	65.86	66.27	66.81	70.25	66.26	1.23	0.812	0.603	0.588
*Bacteroidetes*	26.37	29.25	26.64	19.6	22.74	1.369	0.192	0.08	0.725
*Proteobacteria*	3.65^ab^	2.61^b^	2.52^b^	4.39^a^	4.91^a^	0.29	0.019	0.022	0.023
*Verrucomicrobia*	0.33	0.13	2.78	2.11	2.25	0.714	0.712	0.275	0.672
*Synergistetes*	1.13	0.35	0.22	1.04	0.83	0.173	0.358	0.938	0.158
*Tenericutes*	0.37	0.35	0.38	1.07	0.81	0.099	0.055	0.018	0.821
*Deferribacteres*	0.91	0.35	0.05	0.52	0.9	0.167	0.442	0.905	0.069
*Cyanobacteria*	0.06	0.27	0.09	0.16	0.29	0.049	0.485	0.346	0.835
*Actinobacteria*	0.03	0.03	0	0.08	0.03	0.01	0.203	0.471	0.966
*Lentisphaerae*	0.01	0.01	0.02	0.01	0.04	0.004	0.082	0.03	0.17

Abbreviations: OXA, basal diet with 0% naturally oxidized oil; OXB, basal diet with 25% naturally oxidized oil; OXC, basal diet with 50% naturally oxidized oil; OXD, basal diet with 75% naturally oxidized oil; OXE, basal diet with 100% naturally oxidized oil.

ab Different letters in the same row indicate significant differences (*P* < 0.05), and the same letter means no significant difference (*P* > 0.05).

**Table 10:** Effects of different proportions of naturally oxidized oil on cecal microbiota composition at the genus level of broilers.

Parameter(%)	OXA	OXB	OXC	OXD	OXE	SEM	*P*-value	*P*-linear	*P*-quadratic
*Bacteroides*	15.74	14.05	10.25	8.13	10.98	1.624	0.618	0.201	0.446
*Faecalibacterium*	12.93	13.24	15.36	6.97	5.6	1.768	0.336	0.102	0.35
*Oscillospira*	7.88	5.82	7.27	7.25	8.48	0.404	0.321	0.358	0.138
*Lactobacillus*	2.49^b^	1.36^b^	4.24^ab^	8.03^a^	3.06^b^	0.749	0.038	0.109	0.234
*Ruminococcus*	1.51	1.89	1.9	2.28	2.21	0.143	0.462	0.086	0.662
*Desulfovibrio*	2.45	1.76	1.35	1.8	1.93	0.207	0.605	0.511	0.173
*Ruminococcus*	1.26	2.11	0.92	2.24	2.45	0.202	0.057	0.065	0.428
*Akkermansia*	0.33	0.13	2.78	2.11	2.25	0.714	0.712	0.275	0.671
*Megamonas*	0.42	0.57	0.51	4.59	1.46	0.759	0.375	0.263	0.704
*Alistipes*	0.56	1.81	1.23	1.32	0.6	0.205	0.264	0.77	0.061

Abbreviations: OXA, basal diet with 0% naturally oxidized oil; OXB, basal diet with 25% naturally oxidized oil; OXC, basal diet with 50% naturally oxidized oil; OXD, basal diet with 75% naturally oxidized oil; OXE, basal diet with 100% naturally oxidized oil.

ab Different letters in the same row indicate significant differences (*P* < 0.05), and the same letter means no significant difference (*P* > 0.05).

Lefse analysis was applied to identify the significant differentially abundant ASVs for the entire microbiota at levels from phylum to genus (LDA > 2.0). Lefse's analysis showed that the group without oxidized oil significantly enriched *Ruminobacter*. The group with 25% oxidized oil significantly enriched *Aquabacterium* and *γ-proteobacteria*. 50% oxidized oil group significantly enriched *Bacillales,* and 75% oxidized oil group significantly enriched *Lactobacillus.* Furthermore, the 100% oxidized oil group significantly enriched microbiota such as *Lachnospiraceae, Campylobacterales, Proteobacteria, Helicobacter,* and *clostridium* (Figure 3C).

## DISCUSSION

Lipid peroxidation can lead to the hydrolysis of unsaturated fatty acids, reduce the energy value of feed materials, damage the palatability of feed, and cause harmful effects on poultry health (McGill et al., 2011). Previously, it was found that the FI, weight gain of broiler was decreased, and the FCR was increased by adding oxidized oil (Kishawy et al., 2016). However, Anjum et al. (2002) argued that the low concentration of oxidized oil diet did not affect weight gain and FCR of chicks, and Tan et al. (2018) found that different oxidation levels of soybean oil did not affect broiler growth performance. The current study showed that BWG and FI reached the maximum after adding 50% oxidized corn oil. It may be caused by the low level of oxidized corn oil, which indicates that mild oxidative stress may not be enough to promote the oxidative reaction of broilers but can potentially produce better flavor and improve the palatability of feed. When the oxidized oil is excessive, 100% oxidized oil has no difference with 0% oxidized oil in growth performance, which indicates that the oxidative stress level does not exceed the adaptability of broilers and will not affect growth performance (Liang et al., 2015).

The production of reactive oxygen species (**ROS**) is accompanied by inflammation and apoptosis (Chen et al., 2018). In this experiment, the content of IFN-γ and IL10 in the serum and the mRNA expression of NF-κB, MyD88, Bax in the jejunum of broilers increased significantly after adding 75% oxidized corn oil. A previous study had found that artificially oxidized oil could induce an inflammatory reaction of broilers, and the levels of IL-1β and TNF-α in serum were significantly increased (Dong et al., 2020). At the same time, when a stressor invades, TLRs can recognize specific proteins on the surface of the stressor and activate the NF-κB signaling pathway through adaptor proteins such as MyD88 (Fusella et al., 2020). NF-κB activated by Ikk is transported into the nucleus and induces the expression of a variety of proinflammatory factor genes to play a regulatory role in apoptosis (Li et al., 2015). In addition, Bax promotes apoptosis, and Bcl-2 inhibits apoptosis by preventing the release of cytochrome from mitochondria (Hardwick and Soane, 2013). The increase of proinflammatory factor IFN-γ indicated that the body had immune stimulation because T lymphocytes and natural killer cells would produce IFN-γ after immune and inflammatory stimulation (Boo et al., 2020). IL10 is a potential anti-inflammatory cytokine secreted in the later stages of inflammation. It is an important immunomodulatory pleiotropic cytokine, which is synergistic with proinflammatory factors (Xu et al., 2019), indicating that 75% oxidized corn oil promoted the anti-inflammatory response of broilers. However, there is no difference in inflammatory cytokines and related gene expression between the 100% oxidized corn oil group and the nonoxidized corn oil group. It may be because the inflammatory response is mild by naturally oxidized corn oil, and 100% oxidized corn oil can stimulate the autoimmune regulatory system to clear inflammation (Dokka et al., 2001).

Low immunoglobulin levels are associated with humoral immunodeficiency, and high immunoglobulin levels are associated with inflammation and pathological conditions (Tan and Coussens, 2007). It was found that the IgA, IgG, and IgM content in the mucosa of jejunum were significantly decreased when broilers suffered oxidative stress (Luo et al., 2013). In this study, the serum IgA concentration of the 100% oxidized corn oil group was significantly lower than that of other groups, but the concentration of IgG reached the highest value when 75% oxidized oil was added. The synthesis of immunoglobulin G played a role in activating complement and neutralizing a variety of toxins in the immune response (Chaplin, 2010). The results showed that the immune system of broilers might start to be activated when 75% oxidized oil was added. Furthermore, the decrease of immunoglobulin A content by adding excessive oxidized oil means that the activity of natural killer cells is damaged, which may impact the function of humoral immunity of broilers (Wu et al., 2014).

The intestinal tight junction (**TJ**), composed of tight junction proteins, controls the bypass permeability of intestinal cells (Awad et al., 2017). At the same time, the TJ also plays a vital role in resisting the products of oxidative stress (Tan et al., 2018). The intestinal TJ is mainly composed of claudins, occludin, and zonula occluden-1 (**ZO-1**). The injury of TJ and the abnormal expression of TJ-related genes will lead to the activation of immune cells (Teng et al., 2020). In this study, the mRNA expression levels of occludin and mucin-2 in jejunum were significantly lower than the nonoxidized oil group when 75% oxidized oil was added. It can be speculated that a certain amount of naturally oxidized oil may increase the intestinal permeability of broilers. It might be the consequence for the higher concentration of cytokines in the circulation when 75% oxidized oil added. The TJ genes expression of 100% oxidized corn oil group did not change, is consistent with the results of no changes in inflammatory cytokine that may be caused by the enhancement of the autoimmune system (Soderholm and Perdue, 2001).

Intestinal morphology is closely related to growth performance and intestinal health (Ao and Kim, 2020). The increase of VH of jejunum will increase the surface area of nutrients absorbed, which will have a beneficial impact on the growth of broilers (Calik and Ergün, 2015). The CD of the intestine represents the generation rate of cells, and the shallower the depth of the crypt, the better the maturity of cells (Stamilla et al., 2020). Interestingly, mucin is the main secretion of goblet cells, and the number of goblet cells plays an essential role in maintaining mucosal homeostasis (Birchenough et al., 2015). A recent study found that oxidized soybean oil treatment had no significant effect on the morphological characteristics of broiler ileum, such as the VH, CD, and the density of the neutral/acidic goblet (Dong et al., 2020). In this experiment, the decrease of VH/CD and goblet cells in the jejunum showed that 100% oxidized corn oil changed the original morphology of the jejunum. However, VH of jejunum reached the highest value after adding 50% oxidized oil. If there are many inflammatory cells in the villus, it may cause villus swelling and increase the area of villus, which promotes the absorption of nutrients. So, it may be the reason for growth performance results, indicating that tempered inflammatory reaction maybe beneficial to the growth of broilers and excessive oxidative stress may impair intestinal integrity.

Intestinal ROS is produced by intestinal symbiotic bacteria, which are involved in the regulation of intestinal health (Mishra and Jha, 2019). It had been found that oxidized fish oil could lead to microbial disorder, and the cecal microbial composition of broilers was shifted (Zhou et al., 2021). The current study found a remarkable differentiation of microbial communities among the 5 groups, indicating that feeding naturally oxidized oil changed the intestinal microbial community of broilers significantly. *Proteobacteria* is the most prominent phylum and includes many pathogens, such as *E.coli, Salmonella*, and *Vibrio cholera* (Clavijo and Flórez, 2018). The results suggested that a small amount of oxidized oil might reduce the growth of *Proteobacteria*, but the abundance of *Proteobacteria* enhanced with the increase of oxidative stress. Moreover, the highest relative abundance of *Lactobacillus* was obtained when 75% oxidized oil was added and the previous study had shown that *Lactobacillus* was related to the production of inflammatory cytokine IL22 (Zelante et al., 2013).

Due to the above results, we focused on the dominant microbiota in the 0, 50, and 100% oxidized corn oil groups. LefSe highlights the greater differential abundances of microbiota such as *Ruminobacter* in 0% oxidized corn oil group, *Bacillales* in 50% oxidized corn oil group, and *Lachnospiraceae, Campylobacterales, Proteobacteria, Helicobacter, Clostridium* in 100% oxidized corn oil group. *Ruminobacter* has been shown to degrade polysaccharides and fibers in feed, resulting in a positive correlation between *Ruminobacter* and chicken feed efficiency (Torok et al., 2011). Most *Bacillales* are harmless, and their important characteristic is that they can produce spores with extraordinary resistance to adverse conditions. It was found that *Bacillus subtilis* could effectively colonize the intestinal mucosa and stimulate the growth and development of immune organs in the poultry's digestive tract (Sikandar et al., 2017). Therefore, it could be speculated that a small amount of naturally oxidized corn oil is not harmful to intestinal health. However, a high proportion of *Lachnospiraceae/Streptococcaceae* was known to be associated with metabolic disorders and inflammation in the gut (Zeng et al., 2016). The only known toxin produced by *Campylobacteria* is a cytotoxic distending toxin, which can cause cell cycle arrest and eventually lead to apoptosis of lymphocytes and endothelial cells (Hickey et al., 2005). At the same time, the study found that *Proteobacteria* was prone to colonization in the intestine of IgA deficient mice, which led to persistent intestinal inflammation and increased susceptibility to intestinal injury models (Mirpuri et al., 2014). *Helicobacteria* infection may be involved in the pathogenesis of inflammatory bowel disease by inducing the change of intestinal permeability (Nejabat et al., 2018). *Clostridium* can produce enterotoxins and cytotoxins, which are associated with diarrhea (Simango and Mwakurudza, 2008). Therefore, it could be speculated that microbiota enriched in a high proportion of oxidized corn oil feed might be harmful to intestinal health, resulting in the imbalance of cecal microflora homeostasis. However, there was no sign of inflammatory reaction in the 100% oxidized corn oil group. Taken together, it is contested that the change of cecal microbes may precede the occurrence of inflammation.

## CONCLUSIONS

A low dose of naturally oxidized corn oil is not harmful to the growth of broilers, while a high dose of oxidized corn oil will trigger the inflammatory reaction and have adverse effects on gut health. Therefore, naturally oxidized corn oil should not be added at higher doses in feed production.
